# Removal of Accessory Limb

**Published:** 1889-10-26

**Authors:** 


### REMOVAL OF ACCESSORY LIMB, &c.

Mr. Charles Larkin, assistant lecturer in physiology, University College, and Mr. Robert Jones, honorary surgeon to the Liverpool Hospital, publish an interesting case, headed "Removal of Accessory Limb and Meningocele from the Back of a Child, and its Anatomy."† The patient was an infant four months old, born with a tumour at the back of its neck which had attained to the size of a cocoanut. An oval incision was made round the base of the tumour, leaving sufficient skin to form flaps. The growth was found to be connected with the spinal cord, and the sac was opened, followed by a free discharge of spinal fluid. A thick nervous cord, which looked like a bifurcation of the cord, was divided, and it was then discovered that the laminæ and spinous processes of the lower two cervical and upper two dorsal vertebræ were absent. For the first twenty-four hours the little patient did well, when convulsions came on, causing death. Wood-cuts are given of the tumour, which consisted of two parts—an upper oval part, the meningocele portion, and a lower smaller limb-like portion. The upper portion consisted of skin, tough and strong fibrous tissue, and a cavity. The lower portion contained a scapula and coracoid, a humerus, carpal cartilages, and two fingers.

† *British Medical Journal*, Aug. 10, 1889.

